# Ethical Review of Animal Research and the Standards of Procedural Justice: A European Perspective

**DOI:** 10.1007/s11673-021-10111-5

**Published:** 2021-07-20

**Authors:** Tomasz Pietrzykowski

**Affiliations:** Research Centre for Public Policy and Regulatory Governance, University of Silesia in Katowice, 11b Bankowa str, 40-007 Katowice, Poland

**Keywords:** Law, Ethics, Animal welfare, Procedural justice, Animal experimentation, Research ethics, Ethical committees

## Abstract

Committees established for the ethical review of research involving animals have become a widespread legal standard around the world. Despite many differences in their composition, powers, and institutional settings, they share many common problems related to the well-established standards of procedural justice in administrative practice. The paper adapts the general theory of procedural justice to the specific context of ethical review committees. From this perspective, the main concerns over the procedural aspects of the ethical evaluation of research projects are identified and examined. They include in particular the standards of the committees’ composition, impartiality, fair hearing, appeal, transparency, and benevolence. Their proper reflection in the regulatory regimes of animal ethics committees is necessary to secure the standards of fairness of the ethical review itself. This, in turn, is a condition of the moral and social legitimacy of all administrative and quasi-administrative procedures, including the committees’ operations (irrespective of whether they are legally entrusted with the task of authorizing or only evaluating research projects).

## Introduction

In the contemporary world, an ethical review of animal research vested in independent committees is becoming the widespread standard, at least in developed legal systems. Despite many differences in their composition, powers, and institutional settings, they share many common problems related to the well-established standards of procedural justice in administrative practice. Therefore, it is interesting to inquire into the operation of such committees from the perspective of the general theory of procedural justice. The main context of the inquiry offered in this paper is the European institutional and legal setting of animal ethics committees (AECs). Most of their procedural intricacies seem, however, much more universal. Thus, the argument and findings may be easily adapted to other local contexts.

The paper sets out with a general overview of the institution of AECs. Then, it distinguishes the substantive and procedural aspects of AECs’ review of research projects. The focus of further sections is on the procedural side of AECs’ operation. After summarizing the main tenets of procedural justice theory, the paper discusses from their perspective the following specific issues: the composition of the AEC, the right to fair hearing and appeal, and the requirement of benevolence as well as the transparency of AEC proceedings. The paper closes with some concluding remarks concerning the need for proper balance between the values of fairness and efficacy of AECs’ operation.

## Ethical Review Committees: Where Administration and Ethics Meet

The contemporary system of AECs has its roots in the idea of ethical oversight over medical research on human beings. The idea of establishing independent ethical committees responsible for verifying and approving research projects appeared for the first time in the revision of the Helsinki Declaration on Ethical Principles of Medical Research adopted by the World Health Organization in Tokyo in 1974. It was followed by the development of the institution of ethical committees supervising medical research involving human patients. Over time, such supervision has become a widely recognized and implemented regulatory solution in most parts of the world.

In the 1970s, the first ethical committees on animal experimentation also began to appear. Hereinafter, by “animal ethics committees” or “AECs,” I am referring to the bodies entrusted with the task of the ethical evaluation of research involving living animals. The need for such evaluation is usually restricted to vertebrate animals only, which, according to scientific evidence, are believed to be sentient. In some countries, there are vertebrate species excluded from ethical supervision of AEC, while in the current European law the scope of animals included has been extended to cover also cephalopods (as there is a strong evidence suggesting their sentience, too).

Similarly, as in the case of their human counterparts, there were two principal types of AECs (Rose 2012). Some of them were established as internal bodies of particular research institutes and limited in their scope of operation to the supervision of the research conducted within that specific institute. Committees of this kind have been created since the 1970s in the United States, Canada, and Australia. Some other countries adopted laws giving rise to national and regional AECs responsible for the ethical evaluation of all research conducted in the facilities located in a given area. Such a system was established for the first time in 1979 in Sweden.

An important step forward came when, in 1986, the Council of Europe adopted the Covenant on the Protection of Vertebrate Animals Used for Experimental and other Scientific Purposes. This obliged its parties to ensure “an effective system of control and supervision” over the rules laid down by the convention. In the same year, similar duties were placed on the then member states of the European Community by Council Directive 86/609/EEC (Council Directive 1986). It required that each member state establish an authority to verify the basic legal standards provided for by the directive. Furthermore, the authority was to be notified in advance about every research project involving animals so that it could effectively watch the observance of the principles of animal research in day-to-day laboratory practice.

Since then, both kinds of AECs have spread considerably and today exist in most European countries as well as becoming ubiquitous in American research practice (see Hansen, Goodman, and Chanda 2012). Nonetheless, they still vary considerably in their functions, mode of operation, and powers over the research they supervise (Varga 2013). In Europe, the new directive on the protection of animals used for scientific purposes (EU/63/2010) requires that “an impartial project evaluation independent of those involved in the study should be carried out as part of the authorization process of projects involving the use of live animals.” The provisions of the directive mandate not only that no research project be carried out without prior detailed evaluation and authorization; they also command that such a review must be performed by an independent and impartial authority. Every institution using animals for research must set up an internal animal welfare body to provide advice on the proper conditions of animal use. In addition, all research projects have to be evaluated and approved by an external authority. The directive strongly encourages all twenty-eight member states to establish AECs as bodies competent enough to at least conduct an independent evaluation of such research projects. In some cases, such as in Poland, AECs are authorized not only to evaluate but also to grant or deny formal consent to perform any such project.

Nowadays, at least one such AEC exists in the vast majority of the EU member states, while in most of them, there are also local or regional AECs evaluating research projects on the basis of ethical standards imposed by the directive and relevant national legislation. In some cases, they advise competent authorities, while in others, they evaluate and authorize all research projects involving animals. The committees usually include expert scientists as well as representatives of other stakeholders or the general public (Olsson et al. 2016; Silva et al. 2015).

What is particularly interesting and specific for AECs (alongside the parallel system of ethical committees reviewing research involving human beings) is the very deep intertwining of legal and ethical standards guiding their activities. They are supposed to perform an ethical review but on the basis of the principles and rules specified by the relevant laws. The latter govern both the substantive and procedural aspects of the AEC handling the cases submitted for review. Even if the European legislation is based on a general assumption that most animal research remains, as a matter of principle, indispensable and thus ethically defensible, an AEC may refuse to approve individual projects on ethical grounds. Thus, their decisions have more or less direct—and sometimes far-reaching—consequences for the rights and duties of those who seek to obtain authorization for their research project. It entails that they should be subjected to some form of ultimate judicial control at least in respect to their compliance with the relevant legal standards, in particular pertaining to the procedural aspects of the committee’s proceedings. That brings the committee back to the domain of law through the frame of which the ethical review is to be carried out.

## The 3Rs as the Substantive Standard of Review

Today, the basis for the substantive ethical review of animal experimentation has become known as the 3Rs principle. It was formulated in the 1950s by two British scientists—W.M.S. Russell and R.L. Burch (Russell and Burch 1959)—even if its traces can be found in an early nineteenth century medical treatise by M. Hall (Hall 1831). According to this principle, every research project involving sentient animals ought to be evaluated on the basis of the criteria of replacement, reduction, and refinement. Replacement means that an experiment should be designed in a way to ensure that it obtains its scientific purposes using animals only in cases when they cannot be replaced by any alternative method or means. Furthermore, it should involve as few animals as possible and a species with the least developed sentience (reduction). The refinement requirement is understood as a duty to design an experiment in a way inflicting on animals the least possible pain, suffering, or distress (Chańska and Różyńska 2013).

The approach proposed by Russell and Burch has been widely accepted and turned into ethical policy and legal guidelines for animal research in many places all over the world. In Europe, it has been incorporated into Directive 63/2010 as the basic principles on which the planning and evaluation of projects should rely. According to the directive, the ethical justifiability of an experiment requires

… the selection of the method that is able to provide the most satisfactory results and is likely to cause the minimum pain, suffering or distress. The methods selected should use the minimum number of animals that would provide reliable results and require the use of species with the lowest capacity to experience pain, suffering, distress or lasting harm that are optimal for extrapolation into the target species. (Recital 13)

The 3Rs principle is openly invoked in Article 4 of the directive, where it lays down the obligation of member states to ensure that “wherever possible, a scientifically satisfactory method or testing strategy, not entailing the use of live animals, shall be used instead of a procedure” (replacement). In addition, it is mandatory that “the number of animals used in projects is reduced to a minimum without compromising the objectives of the project” (reduction) and that the “refinement of breeding, accommodation and care, and of methods used in procedures” eliminates or reduces to a minimum the pain, suffering, or distress of the animals involved (refinement). These principles are further developed in Article 13, determining that, when choosing the methods used in the experiment, the one not only using the lowest possible number of animals but also using animals with the lowest capacity to experience pain, suffering, or distress should be preferred.

Pursuant to the current European law and practice, the 3Rs principle remains the important standard for the ethical review of all research projects using sentient animals. Article 38 of the directive provides that the project evaluation should comprise an assessment of its general ethical justifiability. It ought to be carried out in the form of a utilitarian harm-benefit analysis, weighing the expected scientific and social benefits and the likelihood of their achievement with the amount of “costs” in terms of the suffering of the animals involved as well as its conformity to all aspects of the 3Rs principle. In practice, the composition of the committees favours a kind of “peer review” focused on the proper design of the project rather than a more comprehensive, extra-scientific “community review.” The latter would require a prevalence of non-researchers in the composition of the committee as well as extending the review more openly to an assessment of the objectives of the proposed projects if they seem at all doubtful in comparison with the value of animal suffering sacrificed to achieve them (Rippe 2007). In reality, even in AECs with a relatively broad representation of non-researchers, they do not make up the majority. A relevant example can be found in Poland, where the law requires that AECs involve six researchers experienced in animal experimentation, three additional researchers in the field of the social sciences or humanities, and three laypersons representing non-governmental organizations active in animal protection.

Nonetheless, the law specifies the conditions under which the research may be considered ethically justified. However, the application of those substantive criteria to evaluate and authorize projects must be performed within a certain procedural framework. The shape of the latter may materially determine whether the outcome of the ethical evaluation is perceived as legitimate and fair. In some sense, for the overall acceptability of the system of ethical licensing of research projects, it is critical that the process of evaluation itself meets the generally established standards of ethical justifiability. The latter are commonly referred to as “natural” standards of procedural justice or fairness.

## The Problem of Procedural Standards of Justice

The concept of procedural justice or fairness has been developed in legal thought for centuries (Cane 2016). Nonetheless, serious discussion of the theory of procedural justice is rather recent. The *locus classicus* for the modern theoretical account of the concept is John Rawls’s seminal *The Theory of Justice*. The author distinguished the idea of pure, perfect, and imperfect procedural justice. Pure procedural justice implies that there is no independent criterion of a just outcome other than reaching it by means of a just procedure. Alternatively, the idea of perfect procedural justice assumes that there are independent criteria to determine the justice of the outcome, but the procedure guarantees that the correct outcome is actually achieved. The idea of imperfect procedural justice, in turn, entails that procedural conditions do not guarantee the proper outcome but produce the best possible chances of achieving it (Rawls 1999, 74). Meeting the standards of procedural justice seems particularly important in conflicts rooted in inescapably plural ethical attitudes, when, due to value pluralism, it is nearly impossible to find agreement on the substance of the matter in question (Ceva 2008).

Arguably, in practical settings, procedural justice can only be imperfect. Moreover, the very idea of “pure” procedural justice is criticized as incoherent. Discussing the views put forward by Rawls, Sadurski (1995, 51) maintains that “procedural justice is actually derivative from substantive justice. We call certain procedures ‘just’ in so far as we believe that they tend to produce materially just outcomes.” According to this approach, the concept of procedural justice must never be “pure” and fully independent of the substantive justice of the outcomes because the connection between procedural justice and just outcomes is empirical rather than conceptual. Thus, procedural justice—as Sadurski puts it—is neither a necessary nor sufficient condition of substantive justice. Nonetheless, a proper (“just”) procedural framework is the most effective means of making just outcomes as achievable as practically possible. Moreover, a fairly designed procedure may significantly increase the level of the legitimacy of the substantive results for those affected. There is robust evidence delivered by social-psychological research that procedural arrangements respecting the key requirements of procedural justice make people perceive the decisions as more acceptable, irrespective of their substance (Thibaut and Walker 1975; Lind and Tyler 1988).

Thus, the findings of the contemporary procedural justice theories strongly support the intuition present in the history of legal thought from antiquity and encapsulated by Seneca in the following words: “*Qui aliquid statuerit, parte inaudita altera, aequum licet dixerit, haud aequum fecerit”* (He who decides anything without hearing both sides, although he may decide correctly, has by no means acted justly). That is why contemporary law commonly demands that judicial or administrative decisions correspond not only to the substantive standards determined by the relevant rules but stipulates that the legality of a decision depend also on the observance of appropriate procedural standards. The latter are based on the principles traditionally referred to as fairness or natural justice.

## Standards of Procedural Justice in an Administrative Context

The traditional doctrine of natural justice, which is at the heart of contemporary procedural standards, is usually conceived as being composed of two basic requirements—(i) the impartiality of the decision-makers and (ii) a fair hearing for all those who have a legitimate interest in the final outcome. The principle of impartiality is considered to involve rules that exclude a conflict of interests that could cause the decision-maker to be biased or undermine his or her credibility as a neutral umpire capable of deciding the case solely on its merits (Pietrzykowski and Tobor 2003).

To avoid any conflict of interests, the law specifies situations in which a decision-maker is to be held unable (*inhabilis*) to take part in deciding the case and which oblige him to be recused every time there are any other reasons that may compromise his impartiality or call it into question in the eyes of external observers (*suspectus*). The typical sources of bias triggering the duty to recuse include the following:

(i) a personal involvement in the case or a dependence on the outcome (according to the principle *nemo iudex in causa sua*);
(ii) family or professional relations with a party or having a predetermined attitude in regard to the case or any party thereto based on any earlier contacts;
(iii) conflicts or prejudices that may affect the way in which the case is reviewed and the relevant opinions formed.

The second basic requirement of procedural justice is a fair hearing, namely, allowing the interested participants to be heard and to present their views, which are to be taken into account in the decision-making process. It entails appropriate access to information about the facts and evidence that the decision-makers are to consider as well as the right to respond to them and submit additional evidence or information that the participant finds relevant for the case. All this should be done while giving the interested parties reasonable time to act in the proceedings and providing them with sufficient information and guidance or at least allowing them to obtain such guidance from reliable sources.

Apart from these two basic elements of fair procedure, there are several other requirements that should also count as indispensable conditions of procedural justice. These include internal and external transparency. In particular, the procedure must be transparent to the parties, who should be properly informed about the reasons for all decisions taken in their case. It allows them to understand their own situation as well as the motives for the resolutions that may adversely affect their interests. At the same time, the duty to uncover reasons curbs any arbitrariness of the decision-makers and makes it easier to appeal and control the decision. The procedure should also be reasonably transparent externally (for the general public), meaning that it is sufficiently open for the public opinion to build confidence that the decisions are based on considerations of legality, justice, and public interest (Bogucka and Pietrzykowski 2015).

The requirement of transparency is closely linked to the verifiability of decisions, which may also be considered as part of the modern doctrine of fair procedure. It includes mechanisms allowing for the control of all material decisions by courts or other appellate (higher authority) institutions. Additionally, decisions should be reasonably open for independent scrutiny by competent external institutions (such as control agencies) and the public.

The last, but by no means least, condition worth mentioning is benevolence. This notion is understood here as sufficient respect for the legitimate interests of the parties by the procedural arrangements as well as the actual operations of the decision-makers executing those arrangements in practice. To meet the standard of benevolence, the agencies have to provide some level of assistance to the participants in their proceedings. Such proactive assistance may be necessary to help the latter effectively promote their legitimate interests or exercise the rights they hold. In particular, the participants should not be allowed to make detrimental mistakes resulting from insufficient awareness of their procedural situation and the legal framework in which they operate.

It is obvious that all these conditions are closely related to one another and entail numerous more detailed rules and guarantees that contribute to the general quality of the procedural standards applicable in specific cases and types of proceedings.

## Procedural Justice Before Animal Ethics Committees

It is obvious that the procedures applied in AECs raise specific questions that in many cases differ from those typical of general administrative proceedings. Thus, the general standards discussed above need some adaptation and adjustment to address the peculiarities of the review they perform. Moreover, the regulations governing their operations differ in various legal systems. This means that specific procedural problems arise and are resolved under particular rules of applicable law.

Nonetheless, I believe that there is a range of relatively common problems that may be identified and outlined largely irrespective of the details of local legal settings. They are typical for this kind of institution as well as most similar kinds of ethical committees (such as those dedicated to reviewing research involving human beings). They are, obviously, partially related to local laws as well as the respective scientific and administrative culture. Within this scope, the views presented here are offered from a mainly European perspective.

### Composition of the Committees and the Question of a Conflict of Interest

As mentioned above, one of the two fundamental principles of “natural” procedural justice is believed to be impartiality. For a collective body such as an AEC, this extends to all its members and entails the duty to exclude from the panel deciding the particular case anyone who finds him or herself in a conflict of interest. However, the majority of the members of AECs are usually active researchers, often professionally related to the institutions the reviewed projects come from (Schuppli and Fraser 2007; Russell 2012; Johnson 2013). If an AEC is internal to the institution, it is even possible for all its decisions to pertain to projects brought by colleagues and co-workers of the members of the committee. For obvious reasons, this may be conceived as a serious threat to the ability of the committee to impartially evaluate and opine on the projects.

One solution that could be taken into account is reducing the number of AEC members being active researchers (today they usually make up the majority of a committee). This could, however, compromise the AEC’s capability to draw on the easily available professional expertise of its members. Another solution could be to invite external experts or researchers from other institutions to sit on the committee, but this may in some cases create an opposite conflict of interest resulting from the intense competition between scientists affiliated with various institutions and teams working in the same field of research. It would also not eliminate the deeper problem of scientists’ general bias in favour of research. Such bias may be irrespective of any personal ties to the institutions or investigators to conduct it. There might be, however, an opposite bias among the non-scientific activists applying to sit on an AEC, in particular in cases where the public is represented mainly by members of non-governmental organizations engaged in animal protection. Some partial solution mitigating the partiality problem may be to have internal assessments of projects within an AEC assigned to its two members—a scientist reviewing the validity and correctness of the project and a non-scientist highlighting the relevant ethical concerns (Silverman et al. 2017).

Apart from this, the question arises as to the scope of what should count as a conflict of interest in the context of researchers evaluated by their peers sitting on the AEC. It seems plausible to claim that the sole fact of being affiliated with the same institution, or even a personal acquaintance, does not create such a conflict. In order to regard the situation as unacceptable from the perspective of the impartiality principle, it is necessary that a member of the AEC has some special, direct ties to the project or the individuals involved in its preparation or planned performance. They may be of various nature—personal, professional, financial, or family—but have to be sufficiently close to cast doubts on the ability of a given member of the AEC to form an unbiased opinion. Only such grounds disqualify the member as a disinterested, impartial umpire and entail the duty to recuse him from deciding the case.

What is crucial, however, is the rigorous duty to report any alleged conflict of interests so that the AEC may openly deliberate and decide on whether a given relation suffices to recuse a member from taking part in reviewing a given project. The AEC must be made aware upfront of the fact and nature of the relationship between its member and the proposed project. Only under this condition may the relatively narrow scope of what should be regarded as an inacceptable conflict of interest be a sufficient safeguard of the fundamental value of impartiality in the AEC’s decision-making.

### Participation of an Applicant: Fair Hearing and Appeal

The second basic condition of procedural justice is a fair hearing. It is understood as the right of all whose interests may be affected by the outcome of the procedure to present their position and arguments so that they can be taken into account by the decision-making bodies. In the context of an AEC, the main beneficiary of the right to a fair hearing is the researcher applying for a positive opinion or consent to undertake the project. The applicant’s main way of presenting their views to the AEC is via the application process, which involves the applicant justifying the point of the proposal. In many situations, however, the standard of a fair hearing may demand much more than just this initial opportunity for the applicant to make his case before the AEC.

It may happen that the committee finds the arguments submitted in the application unpersuasive and insufficient to accept the project. Arguably, in such a situation, the applicant should have the right to address the doubts or objections of the AEC members. In other words, the application should not be declined without first giving the applicant a chance to respond to the reasons that the AEC is considering as potential grounds to deny the proposal. In addition, it should include the chance to modify, improve, or redesign the project to comply with the conditions (if any) under which the AEC would be ready to find it ethically acceptable. Thus, the respect for the standard of a fair hearing implies that the applicant should be informed about the views of the committee before it takes its final decision, in particular if such views may render the proposal ultimately rejected.

This requires in practice that AECs should proceed not on the basis of the application only, taking their decision without any further involvement of the applicant. To meet the requirements of a fair hearing, the practice of AECs should mandate inviting applicants to a committee session, or postponing a decision that is to be negative where the applicant was not given an opportunity to address the AEC’s concerns. Without implementing such solutions, the procedure may be considered seriously flawed from the perspective of fair hearing standards.

An applicant should also have a right to appeal so that the decision may be reviewed by an independent body to verify potential mistakes resulting in an inaccurate assessment of the project. In practice, it is particularly problematic to find a procedural way to verify the decisions favourable to applicants. Standardly, the right to appeal is vested within the party to the procedure. In the case of an AEC, typically the only party is the applicant. This makes it difficult to have the AEC decision reviewed and corrected in cases when the application is actually accepted despite legal, ethical, or scientific reasons to deny it. This important matter will be briefly addressed further below.

### Transparency

Although it is usually not mentioned in traditional accounts of procedural justice, it seems clear that transparency has today become one of the main standards of all operations of most (if not all) public authorities (Bogucka and Pietrzykowski 2015). This also includes independent committees as long as they are entrusted with the power to decide on the rights and duties of others. This is usually the case with AECs, and the role of transparency to the public and its possible effects on the scope of research considered as ethically defensible is emphasized in the literature (Russell 2012). It is also pointed out that the transparency of animal research is a special case of a wider discourse on science-society relations (MacLeod and Hobson-West 2016). The question of the transparency of their operations has two distinct aspects. The first is the composition of the committee, which may materially affect the level of social control over its workings. The second aspect is the scope of the confidentiality of the information about the projects and the AECs’ deliberations.

As regards the question of AECs’ composition, it is important to allow members of the public to form a part of the committee. The public should be, however, represented not by random laymen totally disinterested in the animal research but rather persons designated by non-governmental organizations active in animal protection. Otherwise, non-researcher members of AECs may be unable to contribute a substantiated ethical argument to the review of advanced scientific projects and hence remain passive and actually ineffective members of the AEC (Schuppli and Fraser 2007). When the public is adequately represented, the diversification of the AEC’s composition has obvious advantages for the scope of considerations and sensitivities playing their part in the AECs’ decisions. Moreover, it may also mitigate the risks of suspicions and mistrust from the side of public opinion. An AEC deprived of this advantage tends to become just one more professional peer-review expert panel guided by the narrow scope of largely uniform, shared values.

The level of transparency is also dependent on the access to the information that is made available to the general public. The scientific community strongly opposes wide access to such information, pointing out the need to protect sensitive data pertaining to the design of projects and innovative research ideas (Holmberg and Ideland 2012). The conflict of those goals and values has been acknowledged in the present EU Directive 63/2010, which reads: “To ensure that the public is informed, it is important that objective information concerning projects using live animals is made publicly available. This should not violate proprietary rights or expose confidential information” (41).

In this way, the directive tries to somehow reconcile considerations of transparency with the confidentiality of information that may have high commercial or scientific value. The solution it lays down provides for a duty to publish “anonymous non-technical summaries of those projects” that should be sufficiently general to remain comprehensible to laymen, preserve the anonymity of researchers, and avoid the disclosure of those details of projects that could compromise the vital interests of researchers or their sponsors.

The regulation is an important step toward striking the proper balance between those contrasting points of view. There is no reason, however, why it should not be supplemented by a right to demand access to the documents produced by the AEC in regard to a particular case by anyone interested. What should be excluded from such access should be, on an exceptional basis, only those parts of the content of the documents that contain confidential information of commercial value. This is the only way to let public opinion be confident that the committees are actually independent, impartial, and diligent guardians of the public interest, taking decisions based on the balancing of all relevant considerations.

Admittedly, such access may increase the risk of unsympathetic public reactions to at least some of the particularly painful projects. Nonetheless, the genuine dialogue between the scientific community and the general public cannot be based on hiding and limiting access to the ways in which animals are actually used in the research. Moreover, it could also help solve one of the main loopholes in the operation of many ethical committees. This is namely the lack of any means to appeal against the committee’s decision if it is favourable to an applicant. Even if the approval of a project is clearly wrong, the researcher is satisfied with the decision. If there are no other parties to the proceedings, there is nobody entitled to appeal the decision.

In practice, there seem to be two possible solutions to this situation. One is the right of each individual AEC member who voted against its decision to initiate its revision by an appropriate body of higher authority. It could be designed analogically to the well-established regulation applied in company law where each shareholder may contest before the court the resolutions adopted by the shareholders’ assembly. A solution of this kind exists in Switzerland although within the legal framework in which the AEC issues an advisory opinion only, but each of its members may challenge the further decision of the respective administrative authority licensing research projects.

An alternative solution could be some external agents, such as animal welfare organizations, being able to appeal or contest AEC decisions. Such a possibility exists currently under Polish law. It allows non-governmental organizations interested in a case being held before an administrative authority to apply for admission as a party to that case. If granted such admission, the organization obtains all the rights of a participant, including the standing to appeal against the final decision.

In effect, in Poland, animal welfare organizations are entitled to request admission to become a party in any case heard before the AEC and thus obtain the right to challenge the final approval of the project before an appellate committee and then in administrative court. There is a paradox, however. Formally, such an organization, until it is actually admitted to the procedure, is not allowed to obtain any information about the relevant case. As a result, a kind of “catch-22” emerges—an organization may receive access to the relevant information only by being admitted to ongoing proceedings, but in order to apply for such admission, the organization needs information it is not entitled to obtain before it is granted the status of a party.

This vicious circle is circumvented in various ways in practice. Nonetheless, it demonstrates that the strict confidentiality of all information about the matters handled by AECs (except for the sole publication of non-technical summaries of the projects, which may take place long after the project has actually been accepted) is an excessive limitation of transparency without any clear justification. A much better and fairer solution is a wider opening of access to information on the issues heard before AECs with severe penalties for anyone who would abuse such access to try to obtain undue advantages for their own scientific or commercial activities.

### Benevolence

Another condition of procedural justice worth mentioning is of a somewhat different nature. It concerns minimizing the risk that the parties to AEC proceedings may suffer any disadvantage due to a lack of knowledge of their own situation and its legal aspects. In other words, an AEC should take utmost care that the legitimate interests of the respective parties to its proceedings are not unfairly affected by its decisions. Therefore, the committee ought to take reasonable care that all interested parties are able in practice to advocate their case and exercise their rights within the proceedings.

This entails the duty to keep the parties duly informed, to reckon with all entities involved or affected by the case, and to avoid imposing on the parties excessive duties or constraints making it difficult for them to effectively pursue their goals. Additionally, the AEC should not neglect the need to explain its decisions, submitting reasons and clarifications—if necessary—as to why some arguments advanced by the parties are unsuccessful. All this is highly instrumental for maintaining and reinforcing trust in the good faith, professional competence, and proper motives of AECs. Improving such trust between the scientific community and public opinion is one of the key benefits that may be brought by the establishment and proper working of ethical committees.

## Concluding Remarks: Fairness vs Efficacy—Towards the Right Balance

The considerations above prove that the procedural justice perspective is a useful and adequate instrument through which to examine and improve at least some deficiencies in the current way AECs are built and operate. Applying standards of procedural justice to AECs widens the scope of the discussion over their model and operation. The predominant view of the AEC focuses on its role in safeguarding substantive ethical standards of animal research. Without prejudice to that end it is necessary, however, to found the AEC itself on an ethically viable basis so that it actually improves the standards of ethics in the scientific community. In particular, the procedural justice approach may materially help in refining the rules and practices of AECs to better address the basic standards of fair procedure.

As with everything else, the concern over standards of procedural justice comes at a price. In this case, the price is mainly the speed and efficacy of the procedure as well as the comfort and ease of the tasks assigned to the AECs’ members. However, it is certainly a price worth incurring. The role of the AEC is to properly balance some of the most vital moral concerns of modern times. On one side is the advancement of science, possibly essential for humans and animals to flourish (even if often much less consequential or in some cases even trivial). On the other is the suffering inflicted on animals, sometimes amounting to extreme suffering that in any other context it would count as the utterly intolerable torturing of sentient creatures.

Finding the proper balance between those values requires diligence and care that can only be achieved by safeguarding appropriate procedural standards. Thus, the significance of designing and complying with at least the basic elements of procedural justice by AECs is twofold. First, it is instrumental to pursue the substantive objectives of their deliberations pertaining to some of the paramount concerns of modern societies. Second, there is some inherent value in following fair procedure. AECs are vested with authority affecting the vital interest of researchers, science, society, and sentient animals. The body entrusted with the right to issue ethical verdicts on such a fundamental domain of social practices most certainly ought to satisfy proper ethical standards itself.
